# A dinuclear zinc complex with (*E*)-4-dimethyl­amino-*N*′-(2-hy­droxy­benzyl­idene)benzohydrazide

**DOI:** 10.1107/S1600536811014462

**Published:** 2011-04-22

**Authors:** Jie Ma, Wei-Zhen Fan, Li-Rong Lin

**Affiliations:** a Department of Chemistry, College of Chemistry and Chemical Engineering, Xiamen University, Xiamen 361005, People’s Republic of China

## Abstract

The title compound, bis­[μ-(*E*)-2-({2-[4-(dimethyl­amino)­benzo­yl]hydrazinyl­idene}meth­yl)phenolato]bis­[formato­zinc], [Zn_2_(C_16_H_16_N_3_O_2_)_2_(CHO_2_)_2_], is a dinuclear Zn^II^ complex containing two Zn^II^ cations, two monovalent anions of a Schiff base ligand, 4-dimethyl­amino-*N*′-(2-hy­droxy­benzyl­idene)benzohydrazide (*L*), and two formate ions. Each Zn^II^ atom chelates with the hy­droxy O atom of salicyl­aldehyde, the imine N atom, the carbonyl O atom, the formate carboxyl­ate O atom and the hy­droxy O atom of the salicyl­aldehyde moiety in a symmetry-related unit. The five-coordinate Zn^II^ atoms form a dimeric centrosymmetric unit with a central parallelepiped Zn_2_O_2_ core and parallel faces derived from the Schiff base ligands. The crystal packing is stabilized by inter­molecular N—H⋯O hydrogen bonds between the amide N atom and the formate carboxyl­ate O atom.

## Related literature

For details of Zn complexes and related applications, see: Shamsipur *et al.* (2001 ▶); Cametti *et al.* (2008 ▶); Winter *et al.* (2009 ▶); Shi *et al.* (2009 ▶); Rai *et al.* (2009 ▶). For potential applications in luminescence materials, see: Erxleben (2001 ▶). For recent advances in biosensory and medicinal therapeutic applications of Zn^II^ complexes, see: Drewry & Gunning (2011 ▶). For other applications of Schiff base–zinc complexes, see: Costamagna *et al.* (1992 ▶); Sunatsuki *et al.* (2002 ▶); Jiang *et al.* (2010 ▶); Li *et al.* (2010 ▶). For details of the synthesis of the Schiff base ligand, see: Pouralimardan *et al.* (2007 ▶). For related literature on zinc complex applications, see: Consiglio *et al.* (2010 ▶); Kwok *et al.* (2004 ▶).
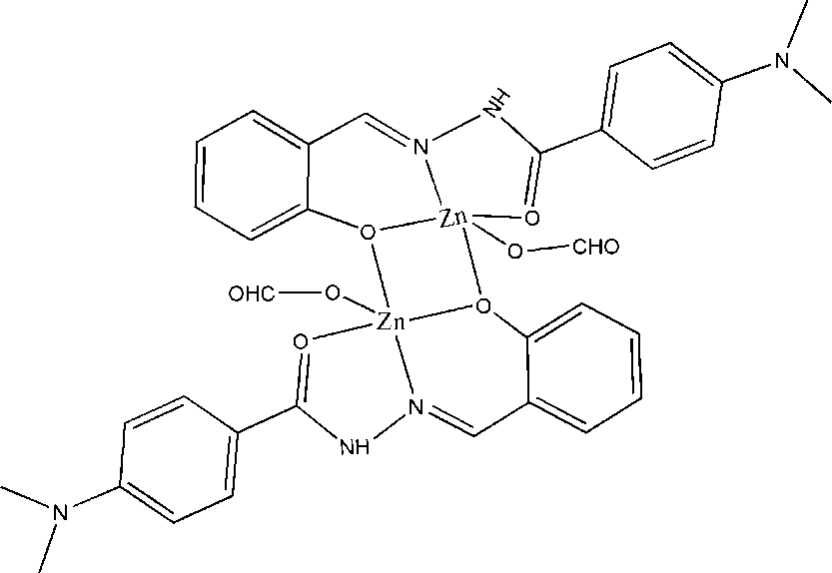


## Experimental

### 

#### Crystal data

[Zn_2_(C_16_H_16_N_3_O_2_)_2_(CHO_2_)_2_]
*M* *_r_* = 785.41
Monoclinic, 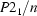

*a* = 14.556 (3) Å
*b* = 6.7607 (14) Å
*c* = 17.085 (3) Å
β = 101.63 (3)°
*V* = 1646.8 (6) Å^3^
*Z* = 2
Mo *K*α radiation
μ = 1.52 mm^−1^
*T* = 173 K
0.10 × 0.10 × 0.08 mm

#### Data collection

Rigaku R-AXIS RAPID diffractometer
Absorption correction: multi-scan (*ABSCOR*; Higashi, 1995 ▶) *T*_min_ = 0.863, *T*_max_ = 0.888
13433 measured reflections
3213 independent reflections
2679 reflections with *I* > 2σ(*I*)
*R*_int_ = 0.032

#### Refinement

*R*[*F* ^2^ > 2σ(*F* ^2^)] = 0.028
*wR*(*F* ^2^) = 0.077
*S* = 1.07
3213 reflections
228 parameters
H-atom parameters constrained
Δρ_max_ = 0.33 e Å^−3^
Δρ_min_ = −0.30 e Å^−3^

### 

Data collection: *RAPID-AUTO* (Rigaku, 2004 ▶); cell refinement: *RAPID-AUTO*; data reduction: *RAPID-AUTO*; program(s) used to solve structure: *SHELXS97* (Sheldrick,2008 ▶); program(s) used to refine structure: *SHELXL97* (Sheldrick, 2008 ▶); molecular graphics: *SHELXTL* (Sheldrick, 2008 ▶); software used to prepare material for publication: *SHELXL97*.

## Supplementary Material

Crystal structure: contains datablocks I, global. DOI: 10.1107/S1600536811014462/jj2088sup1.cif

Structure factors: contains datablocks I. DOI: 10.1107/S1600536811014462/jj2088Isup2.hkl

Supplementary material file. DOI: 10.1107/S1600536811014462/jj2088Isup3.cdx

Additional supplementary materials:  crystallographic information; 3D view; checkCIF report

## Figures and Tables

**Table 1:** Hydrogen-bond geometry (Å, °)

*D*—H⋯*A*	*D*—H	H⋯*A*	*D*⋯*A*	*D*—H⋯*A*
N2—H8⋯O3^i^	0.88	2.00	2.838 (2)	158

Symmetry code: (i) 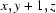.

**Fig. 1.**
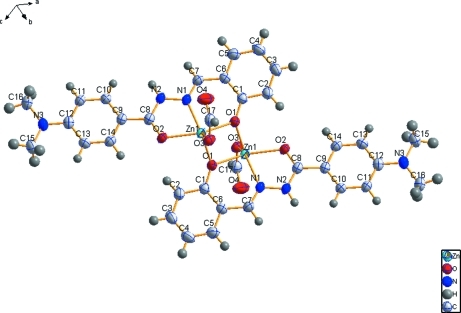
Thermal ellipsoid plot of the title compound at the 30% probability level. Hydrogen atoms are removed for clarity.

## supplementary crystallographic information

### Comment

Zinc(II) Schiff base compounds, which are thermally stable, structurally diverse and easily modified, have attracted great interest due to their potential applications in luminescence materials (Andrea, 2001), biosensory and medicinal therapeutic (Drewry & Gunning, 2011). As part of our ongoing work in the biosensory area, the title Zn-Schiff base complex was synthesized by solvothermal methods and the crystal structure is herein reported. The title molecule consists of two subparallel, tridentate Schiff base ligands, two five coodinate zinc cations and two formyl groups (from the decomposion of dimethylformamide (DMF)). The center plane assumes a parallelogram geometry which is comprised of the alternate atoms of Zn and O atoms (Fig. 1). Crystal packing is stabilized by weak N—H···O intermolecular hydrogen bonds between the amide nitrogen atom and the formate carboxyl oxygen atom (Table 1).

### Experimental

A mixture of ethyl-4-(dimethylamino) benzoate(10 mmol) and hydrazinium hydroxide (50%, 70ml) was stirred at 120 °C for 3h and then filtered. The resulting white residues were recrystallization by ethanol. Then, 2-hydroxybenzaldehyde (2 mmol) was added to the solution of the above powder (2 mmol) in 50 ml ethanol and refluxed for 3h to obtain the crude product. The crude product was purified by recrystallization with the ethanol and dichloromethane mixed solvent(v/v=2:1) to give white solid Schiff base ligand (N-(2-hydroxybenzylidene)-4-(dimethylamino)benzohydrazide, HL) with 85% yield. A DMF solution containing HL (0.2 mmol) and Zn(ClO_4_)_2_^.^6H_2_O (0.1 mmol) was stirred at room temperature for 5h and then was sealed in a 20 ml Teflon-lined stainless-steel autoclave, which was heated to 110^o^ and kept at this temperature for 35h. Yellow block crystals were obtained after the reactor was cooled to room temperature in about 40h.

### Refinement

H atoms were placed at calculated positions with C—H = 0.95 (aromatic), 0.98 (methyl) Å and were refined with *U*_iso_(H)= 1.5 *U*_eq_(C) for methyl H atoms and 1.2 *U*_eq_(C) for aromatic moieties.

### Crystal data

**Table:**

[Zn_2_(C_16_H_16_N_3_O_2_)_2_(CHO_2_)_2_]	*F*(000) = 808
*M**_r_* = 785.41	*D*_x_ = 1.584 Mg m^−^^3^
Monoclinic, *P*2_1_/*n*	Mo *K*α radiation, λ = 0.71073 Å
Hall symbol: -P 2yn	Cell parameters from 11481 reflections
*a* = 14.556 (3) Å	θ = 6.1–55.0°
*b* = 6.7607 (14) Å	µ = 1.52 mm^−^^1^
*c* = 17.085 (3) Å	*T* = 173 K
β = 101.63 (3)°	Block, yellow
*V* = 1646.8 (6) Å^3^	0.10 × 0.10 × 0.08 mm
*Z* = 2	

### Data collection

**Table:**

Rigaku R-AXIS RAPID diffractometer	3213 independent reflections
Radiation source: Rotating anode	2679 reflections with *I* > 2σ(*I*)
graphite	*R*_int_ = 0.032
ω scans	θ_max_ = 26.0°, θ_min_ = 3.3°
Absorption correction: multi-scan (*ABSCOR*; Higashi, 1995)	*h* = −17→17
*T*_min_ = 0.863, *T*_max_ = 0.888	*k* = −8→8
13433 measured reflections	*l* = −21→21

### Refinement

**Table:**

Refinement on *F*^2^	Primary atom site location: structure-invariant direct methods
Least-squares matrix: full	Secondary atom site location: difference Fourier map
*R*[*F*^2^ > 2σ(*F*^2^)] = 0.028	Hydrogen site location: inferred from neighbouring sites
*wR*(*F*^2^) = 0.077	H-atom parameters constrained
*S* = 1.07	*w* = 1/[σ^2^(*F*_o_^2^) + (0.0452*P*)^2^ + 0.0838*P*] where *P* = (*F*_o_^2^ + 2*F*_c_^2^)/3
3213 reflections	(Δ/σ)_max_ = 0.002
228 parameters	Δρ_max_ = 0.33 e Å^−^^3^
0 restraints	Δρ_min_ = −0.30 e Å^−^^3^

### Special details

**Table:**

Geometry. All esds (except the esd in the dihedral angle between two l.s. planes) are estimated using the full covariance matrix. The cell esds are taken into account individually in the estimation of esds in distances, angles and torsion angles; correlations between esds in cell parameters are only used when they are defined by crystal symmetry. An approximate (isotropic) treatment of cell esds is used for estimating esds involving l.s. planes.
Refinement. Refinement of *F*^2^ against ALL reflections. The weighted R-factor *wR* and goodness of fit *S* are based on *F*^2^, conventional *R*-factors *R* are based on *F*, with *F* set to zero for negative *F*^2^. The threshold expression of *F*^2^ > 2sigma(*F*^2^) is used only for calculating *R*-factors(gt) etc. and is not relevant to the choice of reflections for refinement. *R*-factors based on *F*^2^ are statistically about twice as large as those based on *F*, and *R*- factors based on ALL data will be even larger.

### Fractional atomic coordinates and isotropic or equivalent isotropic displacement parameters (Å^2^)

**Table:**

	*x*	*y*	*z*	*U*_iso_*/*U*_eq_	
Zn1	0.604524 (15)	0.57545 (3)	0.527904 (16)	0.03564 (10)	
O1	0.47575 (10)	0.5761 (2)	0.56269 (10)	0.0401 (4)	
N2	0.68239 (11)	0.9705 (2)	0.53175 (12)	0.0381 (4)	
H8	0.6959	1.0948	0.5444	0.046*	
N1	0.60914 (11)	0.8734 (2)	0.55588 (11)	0.0344 (4)	
O2	0.71192 (10)	0.6870 (2)	0.47370 (10)	0.0436 (4)	
C11	0.90778 (14)	1.2366 (3)	0.44114 (14)	0.0418 (5)	
H11	0.9247	1.3710	0.4520	0.050*	
C9	0.80890 (13)	0.9595 (3)	0.45818 (13)	0.0339 (5)	
C12	0.95679 (14)	1.1247 (3)	0.39372 (14)	0.0378 (5)	
C13	0.92810 (15)	0.9259 (3)	0.37917 (15)	0.0419 (5)	
H13	0.9591	0.8446	0.3472	0.050*	
C8	0.73176 (13)	0.8649 (3)	0.48804 (13)	0.0347 (5)	
C6	0.48425 (14)	0.8912 (3)	0.62988 (14)	0.0378 (5)	
N3	1.02861 (13)	1.2013 (3)	0.36293 (13)	0.0476 (5)	
C10	0.83602 (14)	1.1570 (3)	0.47224 (14)	0.0385 (5)	
H10	0.8042	1.2374	0.5039	0.046*	
C1	0.44444 (13)	0.7007 (3)	0.61235 (13)	0.0355 (5)	
C7	0.56179 (15)	0.9695 (3)	0.59851 (15)	0.0414 (5)	
H7	0.5789	1.1034	0.6106	0.050*	
C14	0.85623 (15)	0.8488 (3)	0.41056 (15)	0.0418 (5)	
H14	0.8383	0.7151	0.3993	0.050*	
C15	1.08257 (17)	1.0784 (4)	0.31949 (17)	0.0560 (7)	
H15A	1.1347	1.1554	0.3068	0.084*	
H15B	1.0422	1.0322	0.2699	0.084*	
H15C	1.1074	0.9642	0.3524	0.084*	
C16	1.06386 (17)	1.3985 (3)	0.38441 (18)	0.0547 (6)	
H16A	1.0112	1.4911	0.3788	0.082*	
H16B	1.1058	1.4389	0.3490	0.082*	
H16C	1.0985	1.3987	0.4399	0.082*	
C2	0.37002 (15)	0.6489 (4)	0.64826 (15)	0.0461 (6)	
H2	0.3428	0.5212	0.6387	0.055*	
C3	0.33486 (16)	0.7778 (4)	0.69733 (16)	0.0540 (6)	
H3	0.2835	0.7380	0.7202	0.065*	
C5	0.44609 (16)	1.0176 (4)	0.68003 (16)	0.0504 (6)	
H5	0.4723	1.1458	0.6909	0.060*	
C4	0.37272 (17)	0.9632 (4)	0.71382 (16)	0.0559 (7)	
H4	0.3483	1.0512	0.7479	0.067*	
O3	0.68177 (10)	0.3641 (2)	0.58935 (10)	0.0429 (4)	
O4	0.71583 (15)	0.5810 (3)	0.68721 (13)	0.0722 (6)	
C17	0.72061 (16)	0.4179 (3)	0.65971 (16)	0.0455 (6)	
H17	0.7563	0.3216	0.6933	0.055*	

### Atomic displacement parameters (Å^2^)

**Table:**

	*U*^11^	*U*^22^	*U*^33^	*U*^12^	*U*^13^	*U*^23^
Zn1	0.03384 (15)	0.02593 (14)	0.04950 (19)	−0.00573 (9)	0.01398 (11)	−0.00385 (10)
O1	0.0353 (7)	0.0404 (8)	0.0478 (10)	−0.0092 (6)	0.0162 (7)	−0.0117 (7)
N2	0.0372 (9)	0.0266 (8)	0.0540 (13)	−0.0070 (7)	0.0180 (9)	−0.0025 (8)
N1	0.0336 (8)	0.0281 (8)	0.0432 (11)	−0.0063 (7)	0.0117 (8)	0.0007 (7)
O2	0.0469 (8)	0.0292 (7)	0.0604 (11)	−0.0115 (6)	0.0246 (8)	−0.0085 (7)
C11	0.0446 (11)	0.0323 (10)	0.0517 (15)	−0.0104 (9)	0.0171 (10)	−0.0037 (10)
C9	0.0335 (10)	0.0320 (10)	0.0368 (13)	−0.0043 (8)	0.0084 (9)	0.0010 (8)
C12	0.0349 (10)	0.0399 (11)	0.0396 (14)	−0.0026 (9)	0.0097 (9)	0.0037 (9)
C13	0.0449 (11)	0.0382 (11)	0.0469 (15)	−0.0002 (9)	0.0194 (11)	−0.0060 (10)
C8	0.0329 (9)	0.0319 (10)	0.0389 (13)	−0.0036 (8)	0.0067 (9)	0.0014 (9)
C6	0.0371 (10)	0.0364 (10)	0.0416 (14)	−0.0006 (9)	0.0124 (9)	−0.0042 (9)
N3	0.0465 (10)	0.0476 (10)	0.0548 (14)	−0.0094 (9)	0.0250 (10)	−0.0018 (9)
C10	0.0410 (11)	0.0333 (10)	0.0443 (14)	−0.0055 (9)	0.0159 (10)	−0.0053 (9)
C1	0.0315 (9)	0.0402 (11)	0.0347 (13)	−0.0008 (9)	0.0065 (9)	−0.0020 (9)
C7	0.0427 (11)	0.0305 (10)	0.0536 (16)	−0.0054 (9)	0.0161 (11)	−0.0061 (10)
C14	0.0459 (11)	0.0307 (10)	0.0511 (15)	−0.0066 (9)	0.0150 (11)	−0.0037 (10)
C15	0.0486 (13)	0.0675 (16)	0.0584 (18)	−0.0044 (12)	0.0259 (13)	−0.0030 (13)
C16	0.0475 (12)	0.0561 (14)	0.0650 (19)	−0.0153 (11)	0.0220 (12)	−0.0036 (13)
C2	0.0411 (11)	0.0556 (13)	0.0436 (15)	−0.0127 (10)	0.0138 (10)	−0.0057 (11)
C3	0.0392 (11)	0.0807 (18)	0.0466 (16)	−0.0074 (12)	0.0191 (11)	−0.0078 (13)
C5	0.0490 (13)	0.0467 (12)	0.0576 (17)	−0.0018 (11)	0.0154 (12)	−0.0139 (12)
C4	0.0472 (13)	0.0709 (17)	0.0537 (17)	0.0033 (12)	0.0196 (12)	−0.0178 (13)
O3	0.0489 (8)	0.0299 (7)	0.0510 (11)	−0.0020 (6)	0.0125 (8)	−0.0009 (7)
O4	0.1017 (16)	0.0525 (11)	0.0617 (14)	−0.0037 (10)	0.0148 (12)	−0.0154 (9)
C17	0.0452 (12)	0.0419 (12)	0.0513 (17)	−0.0026 (10)	0.0144 (11)	0.0063 (11)

### Geometric parameters (Å, °)

**Table:**

Zn1—O3	1.9829 (16)	C6—C7	1.444 (3)
Zn1—O1^i^	2.0204 (16)	N3—C15	1.447 (3)
Zn1—N1	2.0681 (17)	N3—C16	1.449 (3)
Zn1—O1	2.0776 (15)	C10—H10	0.9500
Zn1—O2	2.1104 (15)	C1—C2	1.393 (3)
O1—C1	1.339 (2)	C7—H7	0.9500
O1—Zn1^i^	2.0204 (16)	C14—H14	0.9500
N2—C8	1.342 (3)	C15—H15A	0.9800
N2—N1	1.384 (2)	C15—H15B	0.9800
N2—H8	0.8800	C15—H15C	0.9800
N1—C7	1.277 (3)	C16—H16A	0.9800
O2—C8	1.250 (2)	C16—H16B	0.9800
C11—C10	1.374 (3)	C16—H16C	0.9800
C11—C12	1.404 (3)	C2—C3	1.378 (3)
C11—H11	0.9500	C2—H2	0.9500
C9—C14	1.387 (3)	C3—C4	1.375 (4)
C9—C10	1.399 (3)	C3—H3	0.9500
C9—C8	1.470 (3)	C5—C4	1.363 (3)
C12—N3	1.364 (3)	C5—H5	0.9500
C12—C13	1.415 (3)	C4—H4	0.9500
C13—C14	1.371 (3)	O3—C17	1.274 (3)
C13—H13	0.9500	O4—C17	1.206 (3)
C6—C5	1.402 (3)	C17—H17	0.9500
C6—C1	1.419 (3)		
			
O3—Zn1—O1^i^	102.64 (7)	C11—C10—C9	121.2 (2)
O3—Zn1—N1	126.22 (7)	C11—C10—H10	119.4
O1^i^—Zn1—N1	131.13 (7)	C9—C10—H10	119.4
O3—Zn1—O1	107.34 (6)	O1—C1—C2	120.97 (18)
O1^i^—Zn1—O1	78.72 (7)	O1—C1—C6	121.88 (18)
N1—Zn1—O1	85.47 (6)	C2—C1—C6	117.1 (2)
O3—Zn1—O2	95.66 (6)	N1—C7—C6	125.24 (19)
O1^i^—Zn1—O2	102.13 (6)	N1—C7—H7	117.4
N1—Zn1—O2	76.17 (6)	C6—C7—H7	117.4
O1—Zn1—O2	156.30 (6)	C13—C14—C9	122.20 (19)
C1—O1—Zn1^i^	125.90 (12)	C13—C14—H14	118.9
C1—O1—Zn1	128.65 (12)	C9—C14—H14	118.9
Zn1^i^—O1—Zn1	101.28 (7)	N3—C15—H15A	109.5
C8—N2—N1	116.42 (16)	N3—C15—H15B	109.5
C8—N2—H8	121.8	H15A—C15—H15B	109.5
N1—N2—H8	121.8	N3—C15—H15C	109.5
C7—N1—N2	117.73 (17)	H15A—C15—H15C	109.5
C7—N1—Zn1	129.16 (14)	H15B—C15—H15C	109.5
N2—N1—Zn1	112.60 (12)	N3—C16—H16A	109.5
C8—O2—Zn1	114.85 (13)	N3—C16—H16B	109.5
C10—C11—C12	121.76 (19)	H16A—C16—H16B	109.5
C10—C11—H11	119.1	N3—C16—H16C	109.5
C12—C11—H11	119.1	H16A—C16—H16C	109.5
C14—C9—C10	117.34 (18)	H16B—C16—H16C	109.5
C14—C9—C8	118.27 (18)	C3—C2—C1	121.7 (2)
C10—C9—C8	124.35 (19)	C3—C2—H2	119.1
N3—C12—C11	122.44 (19)	C1—C2—H2	119.1
N3—C12—C13	121.0 (2)	C4—C3—C2	121.3 (2)
C11—C12—C13	116.57 (18)	C4—C3—H3	119.4
C14—C13—C12	120.9 (2)	C2—C3—H3	119.4
C14—C13—H13	119.5	C4—C5—C6	122.4 (2)
C12—C13—H13	119.5	C4—C5—H5	118.8
O2—C8—N2	119.51 (18)	C6—C5—H5	118.8
O2—C8—C9	120.87 (19)	C5—C4—C3	118.3 (2)
N2—C8—C9	119.62 (17)	C5—C4—H4	120.8
C5—C6—C1	119.1 (2)	C3—C4—H4	120.8
C5—C6—C7	116.03 (19)	C17—O3—Zn1	112.98 (14)
C1—C6—C7	124.82 (19)	O4—C17—O3	125.4 (2)
C12—N3—C15	121.18 (19)	O4—C17—H17	117.3
C12—N3—C16	120.76 (19)	O3—C17—H17	117.3
C15—N3—C16	117.20 (18)		

Symmetry codes: (i) −*x*+1, −*y*+1, −*z*+1.

### Hydrogen-bond geometry (Å, °)

**Table:**

*D*—H···*A*	*D*—H	H···*A*	*D*···*A*	*D*—H···*A*
N2—H8···O3^ii^	0.88	2.00	2.838 (2)	158.

Symmetry codes: (ii) *x*, *y*+1, *z*.
